# Aspirin Prevention of Colorectal Cancer: Focus on NF-κB Signalling and the Nucleolus

**DOI:** 10.3390/biomedicines5030043

**Published:** 2017-07-18

**Authors:** Jingyu Chen, Lesley A. Stark

**Affiliations:** Cancer Research UK Edinburgh Centre, Institute of Genetics and Molecular Medicine, University of Edinburgh, Crewe Rd., Edinburgh, Scotland EH4 2XU, UK; s1355550@sms.ed.ac.uk

**Keywords:** Aspirin, non-steroidal anti-inflammatory drugs, nuclear factor kappaB, apoptosis, colon cancer, nucleolus, nucleolar, nucleoli, sequestration, stress, RelA, p65

## Abstract

Overwhelming evidence indicates that aspirin and related non-steroidal anti-inflammatory drugs (NSAIDs) have anti-tumour activity and the potential to prevent cancer, particularly colorectal cancer. However, the mechanisms underlying this effect remain hypothetical. Dysregulation of the nuclear factor-kappaB (NF-κB) transcription factor is a common event in many cancer types which contributes to tumour initiation and progression by driving expression of pro-proliferative/anti-apoptotic genes. In this review, we will focus on the current knowledge regarding NSAID effects on the NF-κB signalling pathway in pre-cancerous and cancerous lesions, and the evidence that these effects contribute to the anti-tumour activity of the agents. The nuclear organelle, the nucleolus, is emerging as a central regulator of transcription factor activity and cell growth and death. Nucleolar function is dysregulated in the majority of cancers which promotes cancer growth through direct and indirect mechanisms. Hence, this organelle is emerging as a promising target for novel therapeutic agents. Here, we will also discuss evidence for crosstalk between the NF-κB pathway and nucleoli, the role that this cross-talk has in the anti-tumour effects of NSAIDs and ways forward to exploit this crosstalk for therapeutic purpose.

## 1. Aspirin and Cancer

Incontrovertible evidence from laboratory, clinical and epidemiological studies indicates that aspirin and related non-steroidal anti-inflammatory drugs (NSAIDs) have anti-neoplastic properties and considerable potential as chemopreventative/therapeutic agents [1,2,3,4]. For example, at therapeutic concentrations, NSAIDs induce cell cycle arrest and atypical apoptosis in cancer cell lines [5,6,7,8]. In animal studies, NSAID administration significantly reduces tumour burden in the azoxymethane-induced rat model of colorectal cancer [9]. NSAIDs also reduce tumour burden and increase survival in the multiple intestinal neoplasia (*Min*/+) model of colorectal cancer [10,11,12]. However, in this model tumour burden is mostly affected when mice are exposed to NSAIDs in utero, suggesting the agents act at the early stages of tumour development [13,14]. Meta-analysis of randomised clinical trials (RCTs) for the prevention of vascular disease indicate daily aspirin (75 mg upwards) reduces cancer incidence and mortality. These effects are particularly evident for colorectal cancer where a 30% to 40% reduction in incidence and mortality are observed [15,16]. The risk of developing distant metastasis is also reduced in aspirin users, suggesting a potential benefit for patients with established disease [17,18]. RCTs for cancer prevention indicate aspirin limits recurrence of spontaneous and hereditary intestinal adenomas (the precursor lesion to most cancers). After long term followup, they also indicate aspirin prevents colorectal cancer in (1) women randomised to alternate day low dose (75 mg) aspirin; and (2) patients with Lynch syndrome (the most common type of hereditary colon cancer) [4,19,20,21,22,23]. The most compelling evidence for the chemopreventative effects of NSAIDs comes from epidemiological studies which have consistently demonstrated reduced cancer incidence and improved survival in persons who regularly take aspirin or other NSAIDs [16,24,25,26]. Again, this association is particularly strong for colorectal cancer, with other cancer types showing less consistent risk reduction.

The predominant anti-tumour activity of NSAIDs is recognized to be the selective induction of apoptosis in neoplastic cells [10,27]. However, the mechanisms underlying this pro-apoptotic activity are complex, interconnected, and remain controversial [4,28,29]. In 1982, John R Vane was awarded the Nobel Prize for discovering that aspirin irreversibly acetylates the cyclooxygenase enzymes, thereby blocking the conversion of arachidonic acid to prostaglandins [30]. Cyclo-oxygenase-2 (COX-2 (*PTGS2*)), the inducible form of the enzyme, is frequently upregulated in cancer and together with PGE_2_, is implicated in several aspects of malignant growth including stem cell proliferation, migration, angiogenesis, apoptosis resistance, invasion, and metastasis [31,32,33,34]. Hence, inhibition of COX-2 activity was thought to be the main mechanism for the anti-tumour effects of NSAIDs. Indeed, a body of literature supports this suggestion [35,36,37,38,39]. More recently, it was proposed that aspirin acetylation of COX-1 in platelets, and the consequent inactivation of platelet function, is the only mechanism that can explain the anti-tumour properties of aspirin when taken at low dose [29,40,41]. However, NSAIDs induce cell cycle arrest and apoptosis in colon cancer cell lines that do not express COX-1 or COX-2 enzymes and in mouse embryo fibroblasts that are null for both COX-1 and COX-2 genes [42,43,44]. The growth inhibitory properties of NSAIDs cannot be reversed by addition of prostaglandins [4,9]. Furthermore, NSAID metabolites that do not appreciably affect the catalytic activity of COXs retain their anti-tumor properties in tissue culture [27] and animal models [45,46]. Hence, there is powerful evidence that inhibition of COX is not the only mechanism by which NSAIDs induce apoptosis and prevent the growth of neoplastic lesions [28,47]. A number of COX-independent targets have been identified including the WNT [10,48], AMPK [49,50] and MTOR [51] signalling pathways (reviewed in [4]). In the rest of this review we will focus on the role of nuclear factor-kappaB (NF-κB). In particular, we will examine the evidence for crosstalk between NF-κB signalling and nucleoli in the regulation of NF-κB transcriptional activity and NSAID-mediated apoptosis.

## 2. NF-κB, Cancer and Aspirin

NF-κB is the collective name for a family of ubiquitously expressed, inducible transcription factors that play a critical role in multiple processes including innate and adaptive immune response, inflammation, differentiation, proliferation and survival [52,53,54]. In mammalian cells there are five family members namely, RelA (p65), RelB, c-Rel, p105/p50 (NF-κB1), and p100/p52 (NF-κB2) [55]. These proteins homo- and hetero-dimerize through their Rel homology domain to create a variety of transcription factor complexes [56]. The most common form of NF-κB is p50/RelA heterodimers. In most cell types, this complex exists in the cytoplasm bound to a family of IκB inhibitory proteins (IκBα, IκBβ, IκBγ and Bcl-3). Following cellular stimulation by a plethora of stimuli including cytokines, pathogens, viruses and stresses, IκB proteins are phosphorylated by the IκB kinase (IKK) complex then degraded by the 26S proteasome [57]. Subsequently, NF-κB translocates to the nucleus where it regulates the transcription of target genes including those involved immune function, inflammation, cell adhesion, differentiation, cell growth, and apoptotic cell death.

In healthy cells, a number of feedback mechanisms ensure that activation of the NF-κB pathway is transient [53,56]. However, in chronic inflammatory conditions and cancer, NF-κB is aberrantly active which contributes to disease progression by promoting inflammation, blocking differentiation, driving stem cell proliferation and inhibiting apoptosis [53,54,58].

A substantial body of data supports a critical role for dysregulated NF-κB activity in intestinal tumorigenesis, the cancer type most responsive to aspirin treatment. For example, a recent meta-analysis of expression studies revealed that high expression of NF-κB is significantly associated with late stage colorectal cancer (TNM stage III–IV) and a worse overall 3 and 5-year survival [59]. Transgenic mice with constitutively active IKK in intestinal epithelial cells develop intestinal tumours and show accelerated adenoma development when crossed to *Min*/+ mice [60]. Conversely, inactivation of IKK in intestinal epithelial or myeloid cells attenuates inflammation-associated tumour development [61]. Furthermore, deletion of *RelA* in intestinal epithelial cells prevents formation of adenomas in the *Min*/+ model [62]. These data have identified inhibition of NF-κB activity as a promising therapeutic target for the treatment of this disease.

**Figure 1:**
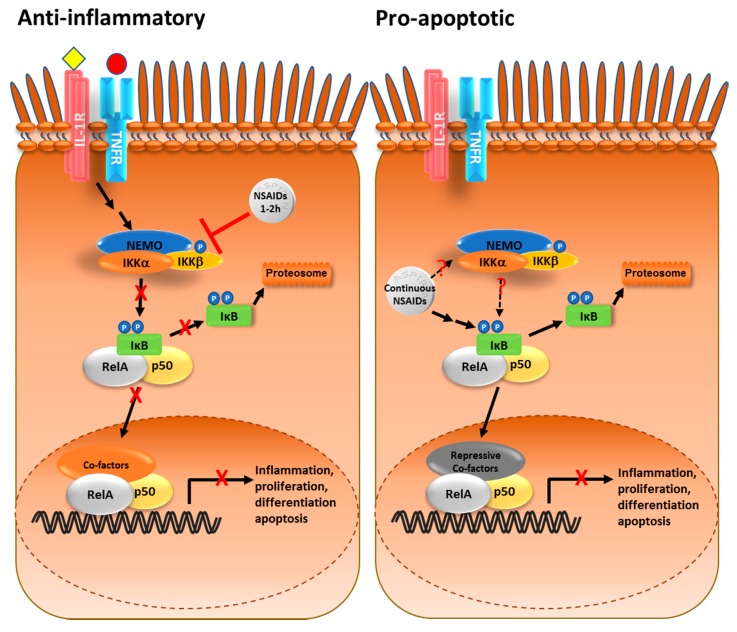
Aspirin modulation of the nuclear factor-kappaB (NF-κB) pathway. (**Left**) The NF-κB transcription factor, most commonly a hetero-dimer of the RelA (p65) and p50 polypeptides, is held in the cytoplasm by the inhibitory protein IκB. When the cell is stimulated by growth factors or cytokines (e.g., interleukin-1 (IL-1) or tumour necrosis factor (TNF)), IκB is phosphorylated by the IκB kinase (IKK) complex, which targets it for degradation by the proteasome. This allows NF-κB to translocate to the nucleus and regulate expression of target genes. In cancer cells, NF-κB is constitutively active which drives tumour progression. Short pre-treatment with aspirin or related non-steroidal anti-inflammatory drugs (NSAIDs) blocks cytokine-mediated activation of the pathway by inhibiting the IKK complex, particularly IKKβ; T bar: NSAIDs inhibit IKK kinase activity. IL-1R: IL-1 receptor; TNFR: TNF receptor; NEMO (IKKγ); (**Right**) In contrast, prolonged exposure to NSAIDs in the absence of additional NF-κB activators stimulates degradation of IκB and nuclear translocation of NF-κB. This NF-κB recruits specific complexes which lead to repression of NF-κB-driven transcription and the induction of apoptosis. Dotted lines: It remains unclear whether the IKK complex plays a role in the stimulatory pathway or whether NSAIDs target IκB by another pathway.

Targeting of the NF-κB pathway by NSAIDs was initially reported by Kopp and Ghosh in 1994, who demonstrated that the aspirin derivative, sodium salicylate, inhibits lipopolysaccharide (LPS) and phorbol 12-myristate 13-acetate (PMA)/phytohemagglutinin (PHA)-mediated degradation of IκB, nuclear translocation of NF-κB and NF-κB transcriptional activity [63]. Yin et al. subsequently demonstrated that salicylate specifically inhibits IKKβ activity in cell lines in vivo and when the agent is added to the kinase in vitro [64]. Since these early publications, NSAID modulation of the NF-κB pathway has been widely reported [28,65]. However, these studies have produced contrasting results dependent upon cell lines and experimental design. In most studies aimed at examining this relationship, cells are treated with NSAIDs for 1–2 h prior to activation of the NF-κB pathway by a potent stimulus (e.g., LPS, Interleukin-1 (IL-1), tumour necrosis factor (TNF)). Under these conditions, NSAIDs block activation of the NF-κB pathway and there is some evidence from in vitro and animal studies to suggest that inhibition of IκB degradation is responsible for the anti-tumour effect of the agents [66,67,68] (Figure 1). However, this experimental design it is entirely inconsistent with the protocol used to demonstrate NSAID-mediated apoptosis of cancer cells, where cells are exposed to the agents for prolonged periods in the absence of additional stimuli [5,6,7,8].

Examination of aspirin effects on NF-κB signalling using this alternative protocol revealed that prolonged treatment of colorectal cancer cells with pharmacologically relevant doses (0.5–5 mM) of aspirin alone actually stimulates the NF-κB pathway, as evidenced by phosphorylation/degradation of IκB and nuclear translocation of RelA [8] (Figure 1). Furthermore, using cells expressing degradation resistant IκB (super-repressor), Stark et al. demonstrated that this stimulation is absolutely required for the pro-apoptotic effects of the agent [8] (Figure 1). Interestingly, stimulation of the NF-κB pathway by aspirin, and the consequent induction of apoptosis, were particularly evident in colorectal cancer cells, which is in keeping with the increased sensitivity of this cancer type to the chemopreventative effects of the agent [7,8]. The NSAIDs diclofenac, sulindac, sulindac sulphone sulindac sulphide, Tolfenamic, indomethacin, celocoxib and ibuprofen, which are all known to protect against colorectal cancer, have also been shown to induce degradation of IκB and nuclear translocation of NF-κB in various cancer cell lines in the absence of additional NF-κB stimuli [69,70,71,72,73,74,75]. Furthermore, in the majority of these studies, NSAID-mediated activation of the NF-κB pathway was causally associated with the induction of apoptosis.

As the above data were generated using tissue culture systems, it was argued that the conditions are not representative of the tumour environment where inflammatory cytokines are abundant. To address this concern, our group examined the effects of aspirin on NF-κB signalling in colorectal neoplasia in vivo, using the HT-29 xenograft and *Min*/+ mouse models. We found that aspirin (at doses resulting in serum salicylate levels relevant to humans (0.5–1.5 mM)) induces phosphorylation and degradation of IκBα, nuclear translocation of RelA and the induction of apoptosis in xenografted HT-29 tumours and in adenomas from *Min*/+ mice [76]. Sulindac sulphide has also been shown to induce degradation of IκB and nuclear translocation of NF-κB in the proximal colons of mice [72]. Furthermore, exposure to low dose (100 μM) aspirin ex vivo was recently shown to stimulate the NF-κB pathway, as evidenced by increased phosphorylation of RelA at serine 536, in 5 of 6 freshly resected, human colorectal tumours [77]. These findings establish that aspirin and other NSAIDs activate the NF-κB pathway in neoplastic epithelial cells in the context of a whole tumour setting, and support the proposition that this effect is important for the anti-tumour activity of the agent.

In reality, NSAIDs likely both activate and suppress activation of the NF-κB pathway in cancer depending on the tumour type and microenvironment. Most solid malignancies require an intrinsic inflammatory response to promote a pro-tumorigenic microenvironment [78]. NSAIDs are thought to act against pre-malignant lesions, at least in part, by altering this response. That is, suppressing pro-tumorigenic immune cell populations while stimulating the adaptive immune system [79]. Notably, colorectal cancer response to NSAIDs is associated with a reduced number of tumour infiltrating lymphocytes [80]. Therefore, it is interesting to speculate that by blocking stimulation of the NF-κB pathway, NSAIDs modulate the tumour microenvironment to reduce the presence of inflammatory cells/cytokines, while stimulation of the pathway in a non-inflammatory environment mediates apoptosis of colorectal cancer cells.

## 3. Crosstalk between the NF-κB Pathway and Nucleoli

As outlined above, several lines of data indicate that NSAIDs stimulate the NF-κB pathway in vitro and in vivo and that this is important for the anti-tumour activity of the agents. However, in most cases, stimulation of the NF-κB pathway by NSAIDs is associated with repression of NF-κB transcriptional activity and downregulation of NF-κB target genes [65,71,75] (Figure 1). In studies aimed at understanding the mechanisms responsible for this repression, a role for crosstalk between NF-κB signalling and the nuclear organelle, the nucleolus, has emerged.

The nucleolus is a highly dynamic, multifunctional organelle [81,82,83,84]. Its main role is in ribosome biogenesis which is the most energy consuming process in the cell and as such, is tightly linked to metabolic and proliferative activity. If cells are exposed to stresses or insults that threaten homeostasis (e.g., Ultraviolet-C (UV-C) radiation, nutrient deprivation, toxic agents), they respond by rapidly downregulating rDNA transcription. This triggers a cascade of nucleolar events that will either allow the cell to repair and regain homeostasis, or, if the damage is too great, undergo apoptosis. Over half of the 4500 proteins found within nucleoli are involved in processes out with ribosome biogenesis e.g., transcription, cell cycle regulation, ubiquitin modification, proliferation and apoptosis [85,86]. These regulatory proteins flux dynamically between this and other cellular compartments depending upon cellular environment [85,87]. While some are released from nucleoli under conditions of cell stress, others translocate to the organelle. For example, NF-κB repressing factor has recently been shown to accumulate in nucleoli in response to heat stress, causing repression of rDNA transcription [88]. P53 and a variety of ubiquitinated proteins accumulate in nucleoli in response to proteasome inhibition, while exposure of cells to heat shock, hypoxia and acidosis causes the accumulation of proteins with a specific nucleolar detention sequence (i.e., von Hippel-Lindau, DNA methyltransferase 1 (DNMT1) and the DNA polymerase subunit POLD1) in nucleolar foci [89,90,91,92,93]. Indeed, nucleolar sequestration of transcription factors and regulatory proteins is increasingly recognised as an important mechanism for controlling gene expression and maintaining cellular homeostasis under stress conditions.

Many proteins known to shuttle through nucleoli are regulators of the NF-κB pathway. For example, the nucleolar protein p14^ARF^, which sequesters MDM2 in the nucleolus to regulate p53 stability, interacts with RelA and inhibits NF-κB-driven transcription [94]. In screens for NF-κB-interacting partners, the predominant proteins identified were the nucleolar proteins NFBP [95] and NPM [96]. The NF-κB regulators NIK (NF-κB-inducing kinase) [97] and NRF (NF-κB repressing factor) [98] also function through nucleolar shuttling. Disruption of nucleolar function is a common denominator for stresses that activate the NF-κB pathway [77]. Furthermore, proteins that have a role in stress-mediated activation of NF-κB reside within this organelle, such as CK2, which forms part of the PolI complex and phosphorylates IκB in response to UV-C [99,100] and EIF2α, that plays a role in NF-κB activation in response to multiple stresses [101,102].

**Figure 2:**
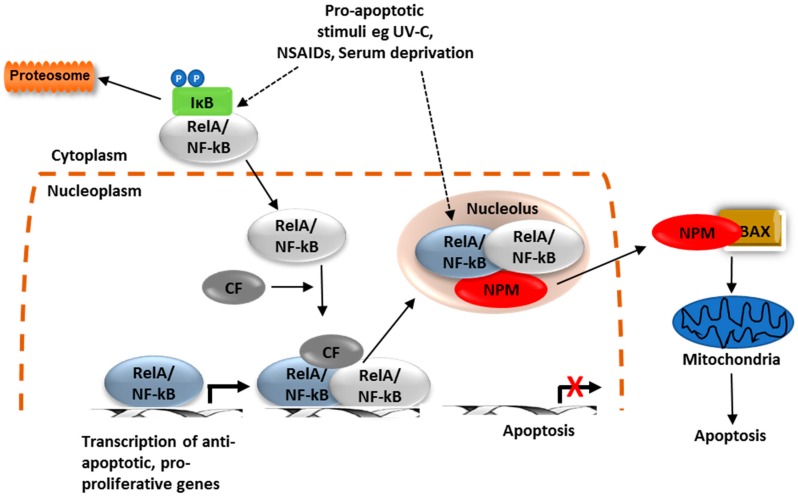
NF-κB-nucleolar crosstalk. Upon exposure of cells to specific pro-apoptotic stimuli, including NSAIDs and chemo toxic agents, IκB is degraded and RelA/NF-κB translocates to the nucleus. This induced NF-κB/RelA recruits specific co-factors (CF)/modifiers that target both constitutive and induced RelA to the nucleolus, reducing basal NF-κB transcriptional activity [62,101]. Once in the nucleolus, RelA induces the relocation of nucleophosmin (NPM) to the cytoplasm which in turn binds to BAX, then transports BAX to the mitochondria to mediate apoptosis [103]. An early response to stresses that induce nucleolar translocation of RelA is disruption of nucleolar morphology, which may “prime” this organelle for nucleolar residency of RelA. Dashed arrows: pathways still under exploration. Solid arrows: published pathways.

When exploring repression of NF-κB-driven transcription associated with stimulation of the NF-κB pathway, it was found that in response to specific pro-apoptotic stress stimuli (e.g., aspirin, serum deprivation and UV-C radiation), the RelA component of NF-κB is sequestered in the nucleolus [65]. A nucleolar localization signal (NoLS) was identified at the N terminus of RelA and, using a dominant-negative mutant with a deletion of this motif, it was shown that nucleolar sequestration of RelA is causally involved in reduced basal NF-κB transcriptional activity and the induction of apoptosis [65] (Figure 2). Importantly, it was found that nucleolar translocation of RelA was absolutely required for the pro-apoptotic activity of aspirin [65,103]. Since this initial study, nucleolar localisation of RelA has been observed in response to the NSAIDs sulindac, sulindac sulphone and indomethacin [71], the naturally occurring derivative of estradiol and antitumor agent, 2-methoxyestradiol (2ME2) [104]; a potent Trk inhibitor and anti-tumour agent, K252a [105]; expression of the homeobox protein Hox-A5 (HOXA5) transcription factor [106], small molecule inhibitors of the CDK4 kinase [107] and the proteasome inhibitors MG132 and lactocystin [103]. In the majority of these studies, nucleolar sequestration of RelA is associated with a decrease in NF-κB-driven transcription. Furthermore, in all studies, it is associated with, or causally involved in, the induction of apoptosis. Nucleolar sequestration of p50 has also been reported. Park et al. demonstrated that the anti-TNF therapy, infliximab, induces “massive” nucleolar localisation of NF-κB/p50 in the hippocampus of rats with a portacaval shunt (PCS). They also demonstrated that this nucleolar localisation is associated with a decrease in transcription of NF-κB target genes and a reduction in neuroinflammation [108].

Given that nucleolar sequestration of RelA causes repression of constitutive NF-κB-driven transcription, it was assumed that the apoptotic effects were mediated through a reduction in transcription of NF-κB regulated, anti-apoptotic genes. However, it was found that once in the nucleolus, RelA triggers a cascade of events that actively promotes apoptosis [109] (Figure 2). That is, nucleolar RelA causes nucleophosmin (NPM)/B23 to relocate to the cytoplasm, bind BAX then transport BAX to the mitochondria to initiate apoptosis [110,111]. Indeed this, and a number of other studies have demonstrated a critical role for both BAX and NPM in the pro-apoptotic effects of NSAIDs [112]. Together, these data identify nucleolar-NF-κB crosstalk as an important regulator of NF-κB transcriptional activity and apoptosis and suggest that this is particular critical for the anti-tumour effects of NSAIDs and chemotherapeutic agents.

As mentioned above, aspirin irreversibly acetylates active site serines to inhibit the activity of cyclooxygenase enzymes. However, as an acetylating agent, it has the ability to acetylate other amino acid side chains [113,114]. Tatham et al. (2017) recently used isotopically labelled aspirin-d3, in combination with acetylated lysine purification and LC-MS/MS, to identify over 12,000 sites of aspirin-mediated lysine acetylation in cultured human cells [113]. Interestingly, gene ontology (GO) analysis indicated that acetylation of nucleolar proteins, including nucleophosmin, was one of the earliest responses to the agent. Immunocytochemical studies also suggest nucleolar morphology is altered as an early response to NSAIDs [65,71], suggesting the intriguing possibility that early effects of these agents on the organelle may enable cross-talk with the NF-κB pathway.

## 4. Conclusions

Despite the overwhelming proof that aspirin prevents colon and other cancers, these agents are still not recommended for cancer prevention in the general population due to their significant side effect profile. Identification of the precise pathway(s) by which daily aspirin inhibits the initiation/progression of cancer is now paramount so that patient populations who may benefit from exposure to the agent can be identified, and safer, more effective alternatives revealed. Inhibition of the cyclooxygenase enzymes, both in platelets and cancer cells, undoubtedly plays a role. However, given that aspirin acetylates many proteins [113,114], other pathways are more than likely involved. Indeed, multiple lines of evidence suggest the agents act in a COX-dependent and independent manner.

There is a consensus in the literature that NSAIDs induce repression of NF-κB-driven transcription, although the pathway to this repression appears to be cell type and context dependent. Nonetheless, given the critical role of de-regulated NF-κB activity in colorectal cancer initiation and progression, it is extremely likely that this repression contributes significantly to the anti-tumour effect of the agents in humans. There are several lines of cross-talk between the prostaglandin and NF-κB signaling pathways and so, it may be that NSAID inhibition of cyclooxygenases inhibits tumour growth through modulation of the NF-κB pathway. In this regard, Chan et al. suggested that NSAID inhibition of COX activity mediates apoptosis not by reducing prostaglandin levels, but by increasing the generation of ceramide, a potent cytotoxic agent that stimulates the NF-κB pathway to induce cell death [115,116]. As more large scale randomised clinical trials are initiated to examine the anti-tumour and chemopreventative effects of aspirin, it will be possible to definitively establish the effects of aspirin exposure on these signalling pathways in human pre-cancer and cancerous lesions, to understand their individual contributions and to determine how they may be interconnected.

Dysfunction of the nucleolus is now regarded a hallmark of cancer as it contributes to tumour growth not only by allowing the protein synthesis required for rapid cell proliferation, but also through de-regulation of critical nucleolar cell growth and death pathways. Hence, modulation of nucleolar function is emerging as an innovative therapeutic strategy. Recent work has uncovered an exciting new role for the nucleolus in the anti-tumour effects of NSAIDs and in particular, cross-talk between nucleoli and the NF-κB pathway. This evolving field is in its infancy and there are still a number of questions to be answered regarding the role of nucleolar sequestration of RelA in the regulation of NF-κB activity and apoptosis in vivo, and how this contributes to the chemopreventative effect of NSAIDs. Identification of the pathways responsible for nucleolar translocation of RelA would allow development of small molecules that act specifically on cancer cells by targeting chromatin bound RelA to nucleoli. Similarly, identification of the apoptotic pathways triggered by RelA within this organelle would allow the development of RelA mimetics that mediate apoptosis by targeting dysfunctional nucleoli. Indeed, further understanding in this area could reveal a whole new class of targets to be exploited for therapeutic purposes.
